# A scRNA-seq Approach to Identifying Changes in Spermatogonial Stem Cell Gene Expression Following *in vitro* Culture

**DOI:** 10.3389/fcell.2022.782996

**Published:** 2022-04-01

**Authors:** Camila Salum De Oliveira, Brett Nixon, Tessa Lord

**Affiliations:** ^1^ Priority Research Centre for Reproductive Science, Discipline of Biological Sciences, The University of Newcastle, Callaghan, NSW, Australia; ^2^ Infertility and Reproduction Program, Hunter Medical Research Institute, Newcastle, NSW, Australia

**Keywords:** spermatogonial stem cell, single cell RNA-seq, *in vitro* culture, spermatogenesis, male fertility

## Abstract

Spermatogonial stem cell (SSC) function is essential for male fertility, and these cells hold potential therapeutic value spanning from human infertility treatments to wildlife conservation. As *in vitro* culture is likely to be an integral component of many therapeutic pipelines, we have elected to explore changes in gene expression occurring in undifferentiated spermatogonia in culture that may be intertwined with the temporal reduction in regenerative capacity that they experience. Single cell RNA-sequencing analysis was conducted, comparing undifferentiated spermatogonia retrieved from the adult mouse testis with those that had been subjected to 10 weeks of *in vitro* culture. Although the majority of SSC signature genes were conserved between the two populations, a suite of differentially expressed genes were also identified. Gene ontology analysis revealed upregulated expression of genes involved in oxidative phosphorylation in cultured spermatogonia, along with downregulation of integral processes such as DNA repair and ubiquitin-mediated proteolysis. Indeed, our follow-up analyses have provided the first depiction of a significant accumulation of ubiquitinated proteins in cultured spermatogonia, when compared to those residing in the testis. The data produced in this manuscript will provide a valuable platform for future studies looking to improve SSC culture approaches and assess their safety for utilisation in therapeutic pipelines.

## Introduction

Spermatogonial stem cell (SSC) function provides the basis for male fertility through self-renewal divisions and the production of progenitor spermatogonia that will differentiate into haploid spermatozoa (Lord and Oatley, 2017). Given the rarity of SSCs within the testis (estimated to be as few as 6,000 cells in adult mice (de Rooij, 2017)), the expansion of this cell population in culture has been an indispensable tool for studying molecular mechanisms that control SSC fate decisions. Indeed, the publication of a methodology with which to maintain SSCs in primary culture in 2004 (Kubota et al., 2004a; Kubota et al., 2004b) provided a platform for the identification of several genes we now know to be essential regulators of SSC maintenance and self-renewal, including *Bcl6b*, *Etv5*, *Lhx1* and *Id4* (Oatley et al., 2006; Oatley et al., 2011).

Beyond their utility for facilitating discovery, primary cultures of undifferentiated spermatogonia also hold potential to substantiate therapeutic approaches for fertility restoration, particularly in childhood cancer survivors. Cryopreservation of immature testis tissue is now actively being offered across the US and Europe to pre-pubertal cancer patients who cannot produce an ejaculate for sperm cryostorage to safeguard their future fertility (Kanbar et al., 2020). Unfortunately, small testicular biopsies are likely to contain few SSCs, thus, it is doubtful that the population captured would be capable of restoring spermatogenesis following auto-transplantation without a prior period of *in vitro* expansion (Gassei and Orwig, 2016). Indeed, transplantation of millions of spermatogonia was required before spermatozoa could be observed in the ejaculate of Rhesus macaques (Hermann et al., 2012). Of note, beyond applicability in clinical practice, the transplantation of SSCs following a period of cryopreservation and *in vitro* expansion also holds promise in the field of wildlife conservation, where similar approaches could be used for endangered species in order to maintain genetically diverse and thus, more robust, populations (Comizzoli, 2015).

Clearly, the ability to robustly maintain SSCs in culture is very valuable. However, although several reports of long-term culture of undifferentiated mouse spermatogonia exist (Kanatsu-Shinohara et al., 2003; Helsel et al., 2017a; Kanatsu-Shinohara et al., 2019), it is well documented that SSC content diminishes with prolonged culture time, while remaining SSCs lose their capacity to regenerate spermatogenesis following transplantation (Helsel et al., 2017a; Kanatsu-Shinohara et al., 2019). Similarly, while many attempts have been made to culture human spermatogonia, these cells have rarely been reported to thrive beyond 40 days (reviewed by Gassei and Orwig (2016)). Incremental advances have certainly been achieved in the culture of mouse spermatogonia, particularly in driving these cells towards a glycolytic pathway by lowering oxygen concentration to 10% (Helsel et al., 2017a) or through over-expression of *Myc* (Kanatsu-Shinohara et al., 2016), however, a temporal decline in SSC activity can still be observed (Helsel et al., 2017a). Despite this, little analysis has been conducted to ascertain the underlying changes occurring to SSCs in culture, although a disruption to metabolism and reduced mitochondrial content certainly appears to be a component (Kanatsu-Shinohara et al., 2019; Lord and Nixon, 2020). In order to ensure the safety of utlising *in vitro*-expanded SSCs for fertility restoration and conservation approaches, and to improve current SSC culture methodologies (particularly in humans), it is important to understand changes that the *in vitro* environment elicits on these cells.

In this Brief Research Report, we provide the first comparative snapshot of gene expression in SSCs from the testis versus those that have been exposed to a period of *in vitro* culture. Using a previously published single cell-RNAseq (scRNA-seq) database of spermatogonia from the adult testis (Hermann et al., 2018), as well as our own previously published database comprised of adult undifferentiated spermatogonia following 10 weeks of *in vitro* culture (Cafe et al., 2021b), we have identified a panel of differentially expressed genes that may be associated with the temporal decline in regenerative capacity observed in culture. Disrupted biological processes identified included expected pathways related to metabolism, particularly oxidative phosphorylation. However, our analysis also identified the dysregulation of previously unexplored biological processes such as ubiquitin-mediated proteolysis and DNA repair. This manuscript will therefore provide a valuable resource for future investigations into the effect of *in vitro* culture on SSC function.

## Materials and Methods

### Animal Ethics Statement

All animal procedures were approved by the University of Newcastle Animal Care and Ethics Committee (ACEC, approval A2019-907). Mice were housed under a controlled lighting regimen at 21–22°C and supplied with food and water *ad libitum*.

### Analysis of scRNA-seq Datasets

Transcriptome analysis was conducted using previously published 10x Genomics Datasets (GSE109033 and GSE163027). Detailed information on the generation of these datasets can be retrieved from the original manuscripts (testis dataset: Hermann et al. (2018), culture dataset: Cafe et al. (2021b)). Briefly, both datasets utilised spermatogonia from mice on a C57BL/6J background. Spermatogonia used to create the ‘culture’ scRNA-seq dataset originated from a *Rosa26-LacZ* mouse line (Jackson Laboratories, stock number 112073), while testis cells were isolated from *Rosa26-LacZ* mice that also carried an *Id4-eGfp* transgene (Chan et al., 2014). These transgenes are utilised as markers for downstream analysis and do not otherwise alter the biology of the spermatogonial population (Chan et al., 2014). Spermatogonia in the adult testis dataset were isolated via fluorescence-activated cell sorting (FACS) (CD9^Bright^/ID4-eGFP+, >2 replicates), and the presence of bona-fide stem cells was confirmed via transplantation analysis (Hermann et al., 2018). Undifferentiated cultures of spermatogonia were initially established using the THY1+ contingent of undifferentiated spermatogonia from the adult testis, however were maintained for 10 weeks (i.e. 10 passages) using “glycolysis optimised” culture conditions (10% O_2_, 5% CO_2_) (Helsel et al., 2017a) before being isolated from feeder cells and prepared for scRNAseq (3 replicates). Stem cell content within cultures was also confirmed using spermatogonial transplantation (Cafe et al., 2021b). It should be noted that we have previously demonstrated that THY1+ selection does indeed capture the ID4-GFP+ contingent of spermatogonia from the adult mouse testis (Lord et al., 2018), thus, although different approaches were used for spermatogonial enrichment in the aforementioned studies, the populations being selected for are largely analogous.

“Culture” and “testis” transcriptomes were imported into Seurat (version 4.03) and merged into a single object (Butler et al., 2018). Low quality cells or doublets with unique feature counts less than 200 or more than 6,500, respectively, or with >25% mitochondrial counts, were filtered from the dataset. Data were normalised using the ‘NormalizeData’ function in Seurat, and were integrated using the ‘Harmony’ algorithm (Korsunsky et al., 2019). The “FindVariableFeatures” function was used to identify variable genes for use in principal component analysis. For clustering and UMAP graphing, 10 significant principal components were used (resolution set to 0.5).

### Gene Ontology Analysis

Unique cluster markers and differentially expressed genes (DEGs) between selected clusters were determined using the “FindAllMarkers” and “FindMarkers” functions in Seurat, respectively. Qualitative analysis of gene lists was conducted using the DAVID Bioinformatics Resources (V6.8) (Huang et al., 2008; Huang et al., 2009) platform.

### SDS-PAGE and Immunoblotting

Immunoblotting was conducted as described previously (Lord et al., 2015). Protein extraction was conducted on the SSC-enriched THY1+ contingent of spermatogonia from a postnatal day 8 testis, or alternatively, the equivalent population of undifferentiated spermatogonia following ten passages of *in vitro* culture (Cafe et al., 2021b). Postnatal day 8 testes were used for this purpose to avoid contamination by elongating spermatids, which often elute from the MACS column following THY1+ spermatogonia selection from the adult testis and cannot be excluded from immunoblotting analyses as they can with scRNA-seq. Protein extraction was achieved *via* a 5 min incubation at 100°C in SDS extraction buffer (2% [w/v] SDS and 10% [w/v] sucrose in 0.1875 M Tris, pH 6.8) containing a protease inhibitor cocktail (Roche). Lysates were resolved on a 4–12% polyacrylamide gel (BioRad) and transferred to a nitrocellulose membrane using standard techniques. Membranes were blocked in 3% bovine serum albumin (BSA) diluted in Tris-Buffered Saline with 0.1% Tween (TBST). Primary antibodies utilised were anti-ubiquitin (Thermo Fisher Scientific, MA1-10035, 1:1000) and anti-*α*-tubulin (T5168, Sigma Aldrich, 1:4000). Following an overnight incubation in primary antibody (diluted in 1% BSA/TBST), membranes were washed 3 times in TBST and incubated in goat anti-mouse IgG HRP (1/10,000) for 1 h at room temperature. Blots were developed using ECL reagent (GE Healthcare) an imaged on a ChemiDoc Imaging System (BioRad). Densitometric analysis was conducted using the public sector image-processing program ImageJ (version 1.52A; National Institutes of Health), and values were normalised to the loading control (tubulin).

### Statistical Analysis

Analysis of scRNA-seq datasets is described in detail above. All additional experiments were conducted using a minimum of three biological replicates. Quantitative data are presented as mean ± SEM. Differences between means was determined statistically using the *t*-test function of GraphPad Prism 9 software. A value of *p* < 0.05 was considered to be statistically significant.

## Results

### scRNA-seq Analysis of a Merged Dataset Containing Undifferentiated Spermatogonia From the Testis and Following *in vitro* Culture

**FIGURE 1:**
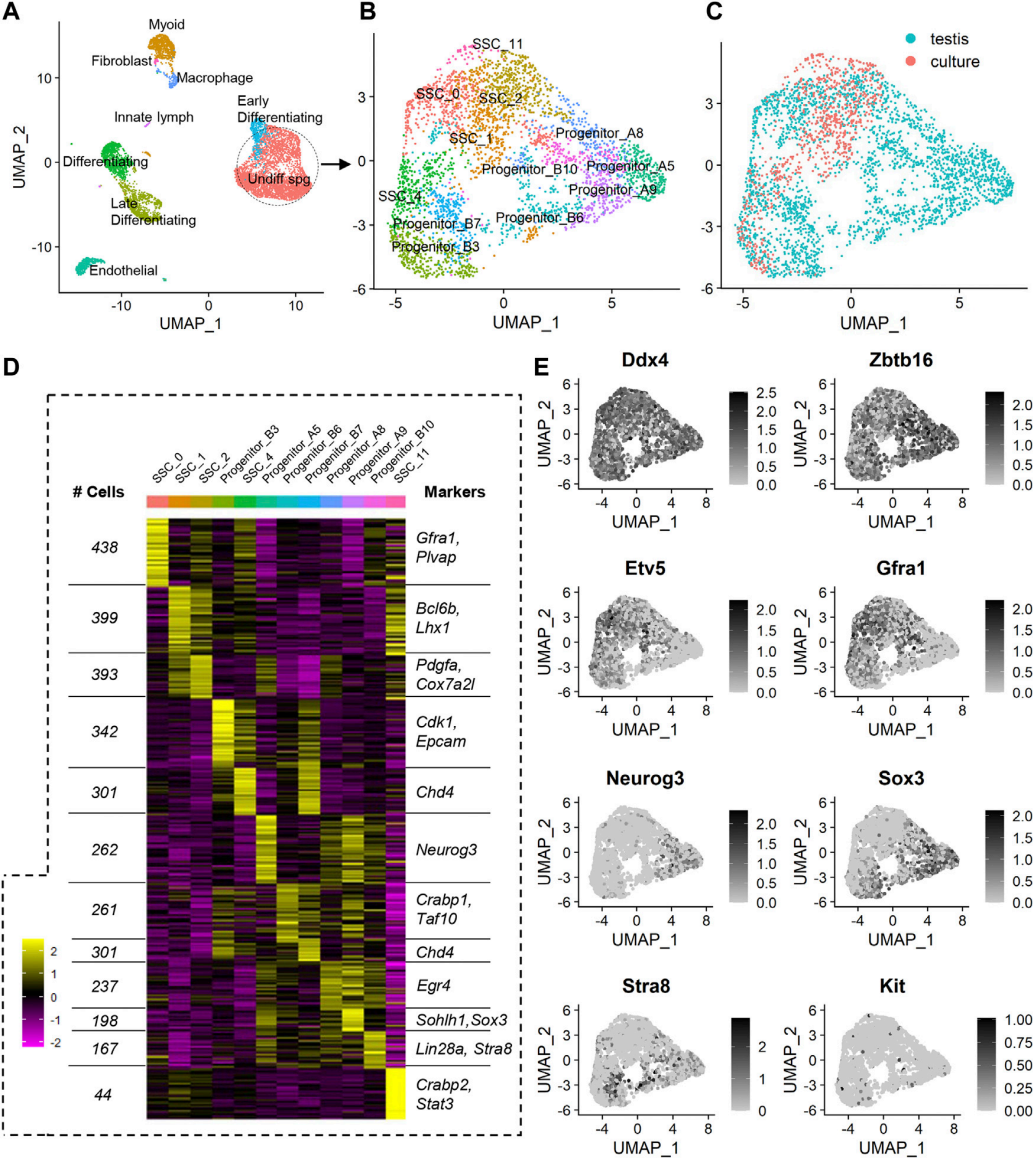
scRNA-seq analysis of a merged dataset containing undifferentiated spermatogonia from the testis and following *in vitro* culture. **(A)** Unsupervised clustering analysis of a merged dataset containing adult mouse testis cells (enriched for spermatogonia) and undifferentiated adult mouse spermatogonia that had been subjected to 10 weeks of *in vitro* culture. The dataset was projected onto UMAP plots and nine distinct cell populations were identified based on expression of known markers. **(B)** In order to determine the effects of *in vitro* culture on undifferentiated spermatogonia specifically, somatic cells and later stage germ cells were excluded from downstream analyses. Re-clustering of undifferentiated spermatogonia and projection onto UMAP plots revealed 12 populations. **(C)** UMAP plot depicting localisation of cells from testis and culture datasets. **(D)** Heat map showing effective clustering, the number of cells in each cluster, and markers associated with each cluster (see also Supplementary Dataset S1). **(E)** Feature plots showing expression of known markers for germ cells (*Ddx4*), undifferentiated to early differentiating spermatogonia (*Zbtb16*), SSCs (*Etv5*, *Gfra1*), progenitors (*Neurog3*, *Sox3*), and differentiating spermatogonia (*Kit*, *Stra8*).

In order to assess how periods of *in vitro* culture influence gene expression in the undifferentiated spermatogonia population, a scRNA-seq comparison was conducted using previously published datasets comprised of adult mouse spermatogonia (Hermann et al., 2018) (from herein referred to as the “testis” dataset), and our own undifferentiated adult mouse spermatogonia that had been subjected to 10 weeks of *in vitro* culture (i.e. passage 10) (Cafe et al., 2021b) (herein referred to as the “culture” dataset). A merged dataset was created using Seurat (Butler et al., 2018), which contained 6,650 cells from the testis (enriched for spermatogonia but also containing some testicular somatic cells), and 784 undifferentiated spermatogonia from culture (Figure 1A; Supplementary Figure S1A). Each cell from the testis dataset had an average of 12,908 unique molecular indices (UMIs) and 3,840 median genes per cell, while the culture dataset had an average of 19,193 UMIs and 4,255 median genes per cell.

In order to negate batch effects, the Harmony algorithm (Korsunsky et al., 2019) was utilised for integration of the two datasets. This method was selected based on previously published comparative studies that reported superior outcomes using Harmony, when compared to other data integration approaches (Tran et al., 2020). Indeed, following Harmony integration, it could be appreciated that undifferentiated spermatogonia from the ‘culture’ dataset aligned with the undifferentiated spermatogonia from the ‘testis’ dataset (Supplementary Figure S1A), creating a population that was distinct from the differentiating germ cells and somatic cells, as expected (Figure 1A; Supplementary Figure S1A).

Unsupervised clustering projected onto Uniform Manifold Approximation and Projection (UMAP) analysis plots revealed 18 populations in the Harmony integrated dataset (Supplementary Figure S1B). Based on the expression of known markers for spermatogonial sub-populations and somatic cells in the testis (which were used to classify cell populations in the original ‘testis’ dataset (Hermann et al., 2018), and in other previously published mouse testis datasets (Law et al., 2019)), identities were assigned to these 18 clusters, resolving nine broader populations of cells: undifferentiated spermatogonia (encompassing four sub-populations of cells on the spectrum from SSC to progenitor spermatogonia, see Supplemental Dataset one and Supplementary Figure S1C for marker expression), early differentiating spermatogonia, differentiating spermatogonia, late differentiating spermatogonia/early spermatocytes, peritubular myoid cells, endothelial cells, macrophages, innate lymph, and a fibroblast cell cluster that is primarily comprised of SNL 76/7-4 mouse embryonic fibroblast feeder cells that were carried through in the ‘culture’ scRNAseq dataset (Figure 1A). A complete list of cluster markers is provided in Supplemental Dataset 1, and feature plots depicting expression of a subset of these cluster markers are provided in Supplementary Figure S1C.

Given the discrepancy between cell numbers in the original testis and culture datasets, and the known capacity for such discrepancies to create challenges for efficient data integration and clustering (Forcato et al., 2020), we elected to focus on the undifferentiated spermatogonia population specifically (circled in Figure 1A), given that differentiating spermatogonia and somatic cells are not a component of the ‘culture’ dataset and are not of interest for addressing the specific aims of this manuscript. Re-clustering of the Harmony-integrated undifferentiated spermatogonia population was performed Supplementary Figure S2A), revealing 12 populations of cells (Figures 1B, C). Effective clustering was confirmed via heatmap analysis (Figure 1D), and a subset of cluster markers (and the number of cells per cluster) are listed in Figure 1D. A complete list of unique cluster markers is provided in Supplementary Dataset S1. To assign identities to spermatogonial sub-populations, expression of known SSC markers (*Etv5, Bcl6b,* and *Gfra1*), progenitor markers (*Sox3* and *Neurog3*), and markers of differentiation (*Kit* and *Stra8*) were assessed (Figure 1E; Supplementary Figure S2B; Supplementary Dataset S1) (Hermann et al., 2018). Based on the elevated expression of SSC markers, and absence of expression of progenitor and differentiating spermatogonia markers, clusters 0, 1, 2, 4 and 11 were categorised as SSCs (Figure 1E; Supplementary Figure S2B). It was noted that progenitor spermatogonia fell in to two ‘sub-categories’ in which they either displayed dual expression of *Sox3* and *Neurog3* (denoted ‘Progenitor A’, capturing clusters 5, 8 and 9), or expressed *Sox3* only, along with increased expression of *Stra8* (denoted ‘Progenitor B’, capturing clusters 3, 6, 7 and 10) (Figure 1E; Supplementary Figure S2B). This categorisation aligns with SOX3+/NEUROG3+ and SOX3+/NEUROG3- subsets of progenitors that have been previously identified in the testes using antibody-based techniques (Nakagawa et al., 2021).

**FIGURE 2:**
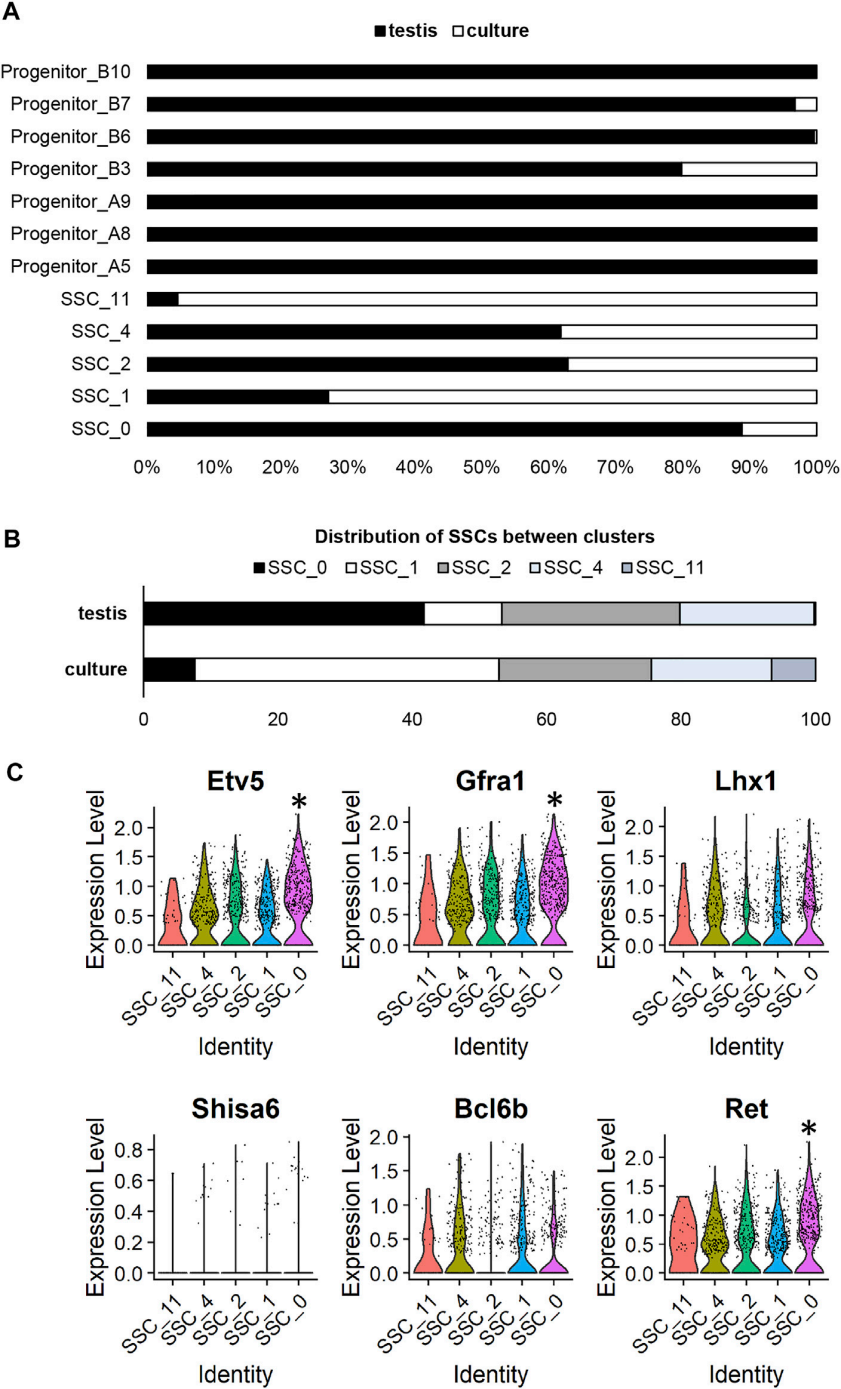
Assessing cluster distribution and expression of self-renewal genes in undifferentiated spermatogonia from culture versus testis. **(A)** Composition of each cluster based on dataset origin (culture versus testis). **(B)** A comparison of the distribution of SSCs from the testis and culture datasets. **(C)** Violin plots depicting expression of genes previously reported to be involved in SSC maintenance. (* represents a significant difference at *p* < 0.001).

In viewing UMAP plots that depict the dataset origins of each cell, it was clear that although the undifferentiated spermatogonia do largely congregate together, there are a number of clusters that are preferentially occupied by cells from either testis or culture datasets. For instance, 90% of cells in cluster SSC_0 originated from the testis dataset, whereas >95% of cells in SSC_11 and >70% of cells in SSC_1 originated from the culture dataset (Figures 1C; Figure 2A). Also interesting was the observation that progenitor spermatogonia from culture only occupied the ‘B’ sub-class (predominantly cluster Progenitor_B3), while progenitors from the testis occupied both ‘A’ and ‘B’ clusters ((Figures 1C; Figure 2A). In focusing on the distribution of SSCs specifically amongst clusters; >40% of testis SSCs occupied the SSC_0 cluster, followed by 27% in SSC_2, and 19% in SSC_4 (Figure 2B). For SSCs from culture, >45% occupied the SSC_1 cluster, followed by 23% in SSC_2, 18% in SSC_4 (Figure 2B).

As a whole, the expression of a broad range of SSC markers (*Etv5*, *Gfra1*, *Lhx1*, *Bcl6b, Ret*) was robust across all SSC clusters (Figure 2C), noting that expression of *Shisa6* (Tokue et al., 2017) was below levels of detection (Figure 2C), and *Id4* levels could not reliably be assessed given that spermatogonia from the testis, but not from culture, came from an *Id4-eGfp* transgenic mouse line (Helsel et al., 2017b), with transgene expression causing an artefactual increase in expression in the testis dataset (Martin and Wang, 2011). It is interesting to note, however, that cluster SSC_0 exhibited a small but significant (*p* < 0.001) increase in expression of a subset of genes that have been reported to play a role in SSC maintenance, including; *Etv5* (Wu et al., 2011), *Gfra1* (He et al., 2007), *Ret* (He et al., 2007), *Sall4* (Chan et al., 2017), *Chd4* (Cafe et al., 2021b), and *Plvap* (Nakagawa et al., 2021) (Figure 2C; Supplementary Dataset S1). In considering that 49 spermatogonia from culture (representing 6.8% of total cells from the culture dataset) reside within the SSC_0 cluster, one could postulate that these may be the SSCs that colonise recipient testes post-transplantation. Indeed, when cells from these cultures were transplanted, they produced an average of 270 colonies per 100,000 cells (Cafe et al., 2021b); equating to ∼2.2–5.4% of cells possessing regenerative capacity when factoring in a colonisation efficiency of 5–12% (Nagano et al., 1999; Ogawa et al., 2003).

### Identifying Changes to Gene Expression Occurring in Undifferentiated Spermatogonia Following a Period of *in vitro* Culture

**FIGURE 3:**
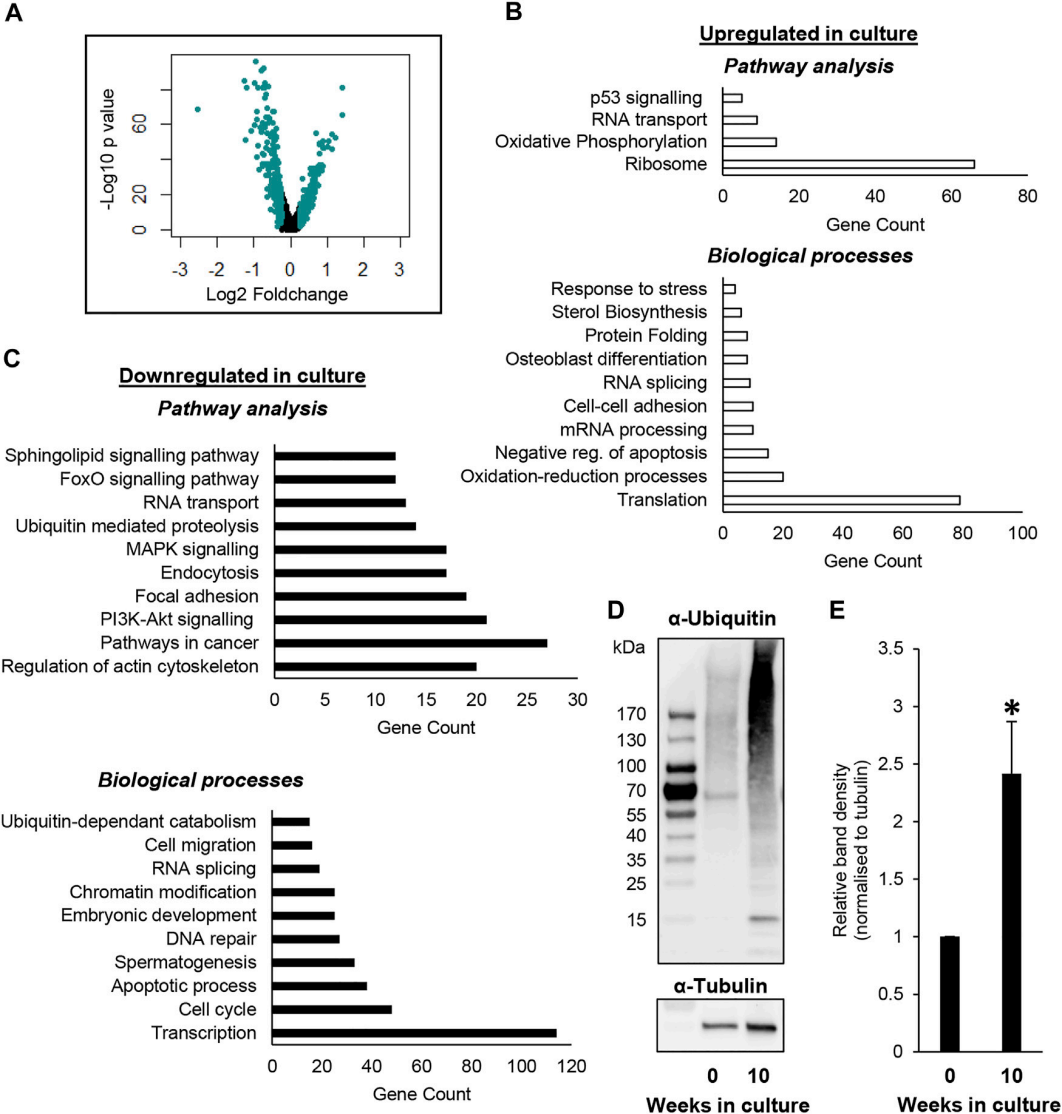
Identifying DEGs between undifferentiated spermatogonia from culture versus the testis. **(A)** Volcano plot depicting Log2 foldchange and –Log10 *p* value of DEGs identified in a comparison of ‘SSC_0’ (testis predominant) and ‘SSC_1’ (culture predominant) clusters. **(B)** Analysis identifying pathways and biological processes that are upregulated in SSCs from the SSC_1 cluster. **(C)** Pathways and biological processes that are downregulated in the SSC_1 cluster. For outputs from DEG analysis see also Supplemental Dataset 1. **(D)** Immunoblot depicting increased levels of ubiquitinated proteins in undifferentiated spermatogonia following 10 weeks of *in vitro* culture, as compared to the equivalent SSC-enriched (Thy1+) population taken directly from the testis (i.e. passage 0/week 0). **(E)** Densitometry analysis demonstrating a significant increase in ubiquitinated proteins in undifferentiated spermatogonia following 10 weeks of *in vitro* culture (*n* = 3 biological replicates, *p* < 0.05).

To gain further understanding on the effects that *in vitro* culture may be eliciting on undifferentiated spermatogonia over time, and thus mechanisms that may underpin the temporal loss of SSCs (Helsel et al., 2017a), a list of DEGs was generated using the ‘FindMarkers’ function in Seurat, comparing the ‘SSC_0’ and ‘SSC_1’ clusters, given that these were predominated by SSCs from the testis and culture datasets, respectively. Using this strategy, 924 DEG’s were identified with an adjusted *p* value < 0.05 and a Log2-foldchange value > 0.25 (Figure 3A; Supplementary Dataset S1). Among these DEGs, 644 were downregulated in SSC_1, while 280 were upregulated. Gene ontology (GO) analysis was performed to identify enriched biological processes and pathways within these gene lists. A number of key themes were identified in genes that were upregulated in the ‘SSC_1’ cluster predominated by cells from culture, including oxidative phosphorylation (e.g. *Cox6a1*, *Cox7C*, *Ndufb7*), protein folding (e.g. *Hsp90ab1* and *Hsp90b1*), and sterol biosynthesis/lipid metabolism (e.g., *Cyp51*, *Hmgcr*) (Figure 3B; Supplementary Dataset S1). Biological processes and pathways that were enriched in the list of downregulated DEGs included chromatin modification (e.g., *Chd4*, *Chd7*)*,* ubiquitin mediated proteolysis (e.g., *Psmd4*, *Psmd2*), spermatogenesis (e.g., *Tex19*, *Sycp1*), and DNA repair (e.g. *Rad51*, *Brca2*) (Figure 3C; Supplementary Dataset S1).

Finally, to demonstrate the utility of our dataset, we utilised an independent set of undifferentiated spermatogonia populations to provide ‘proof-of-concept’. These populations were again isolated either directly from the testis (THY1+) or from primary cultures of spermatogonia that were established from the THY1+ population and maintained for a period of 10 weeks. Given that ‘Ubiquitin mediated proteolysis’ and ‘Ubiquitin-dependent catabolism’ were identified to be downregulated in culture (Figure 3C; Supplementary Dataset S1), we elected to establish whether there is indeed impaired clearance of ubiquitinated proteins by the proteasome in cultured spermatogonia (Figures 3D, E). Immunoblotting analysis using an *α*-ubiquitin antibody revealed a striking accumulation of ubiquitinated proteins within lysates from cultured spermatogonia. This was particularly the case for proteins of a higher molecular weight (>75 kDa), albeit, an increase in ubiquitination was also evident in low molecular weight proteins (Figure 3D). Densitometry analysis was performed on the entire complement of proteins, identifying a statistically significant >2-fold increase of ubiquitinated proteins in cultured spermatogonia (Figure 3E, *p* < 0.05, *n* = 3, normalised to tubulin). These results clearly demonstrate the biological relevance of the scRNA-seq dataset we have generated, which has identified disruption of several integral pathways for cellular function and longevity and thus provides a platform for future investigation into adapting cell culture conditions for safe, long-term maintenance of SSCs.

## Discussion

In addition to being the reservoir that fuels ongoing male fertility, SSCs hold considerable therapeutic potential across multiple species, with their utility spanning from fertility restoration in a clinical context (Gassei and Orwig, 2016), to biobanking for conservation of endangered species (Comizzoli, 2015). However, the rarity of this cell population in the mammalian testis means that the adoption of SSC-based technologies is likely to be reliant on the success of *in vitro* expansion. As with any cell type, exposure of SSCs to an *in vitro* environment inevitably instigates biological changes, in this case, resulting in a diminishment of their regenerative capacity (Helsel et al., 2017a; Kanatsu-Shinohara et al., 2019). In this manuscript, we have identified a suite of differentially expressed genes between undifferentiated spermatogonia retrieved from the testis, versus those maintained in primary culture for a period of 10 weeks. This analysis identified the dysregulation of pathways such as metabolism and DNA repair and formed the basis of seminal findings that suggest that proteasomal degradation of ubiquitinated proteins becomes dysfunctional with increasing culture time. Data produced in this Brief Research Report provide a critical platform to assess the safety of utilising cultured SSCs for therapeutic purposes and highlight the need to further adapt culture conditions to more closely resemble that of the endogenous niche.

Although our data identify differential gene expression between SSCs from the testis versus culture, it is firstly important to note that a majority of SSC signature genes, such as *Etv5*, *Gfra1*, *Lhx1*, *Bcl6b* and *Ret*, retain high levels of expression in cultured cells at the 10 week time point analysed (Figure 2C). This aligns with the continued capacity for a portion of the cells to regenerate spermatogenesis upon transplantation into a recipient testis (Cafe et al., 2021b) and demonstrates the value of these primary cultures for exploratory experiments into SSC function, particularly at early time points/passage numbers. This is in contrast to the immortalised GC-1 ‘spermatogonia’ cell line (Hofmann et al., 1992), for which transcriptome comparisons to scRNAseq data produced from mouse testis have demonstrated little resemblance to spermatogonia or spermatocytes, and instead a gene expression signature that most closely resembles somatic cells within the testis (Norman et al., 2021).

In assessing differentially expressed genes between testicular and cultured spermatogonia, a disruption to metabolism-regulating genes was readily identified. Specifically, Gene Ontology analysis identified ‘Oxidative phosphorylation’ and ‘Oxidative-reduction processes’ as pathways that were upregulated in undifferentiated spermatogonia from culture. This group of genes included *Cox6a1* and *Ndufb7*; components of the mitochondrial electron transport chain that are integral for aerobic metabolism. Given that multiple independent groups have demonstrated that driving glycolysis in spermatogonial culture significantly improves SSC maintenance (Kanatsu-Shinohara et al., 2016; Helsel et al., 2017a), it would be intuitive that prolonged culture time causes a shift towards oxidative phosphorylation that is consequently associated with the observed decline in regenerative capacity (Helsel et al., 2017a). Indeed, this is a hypothesis that we have put forth previously (Lord and Nixon, 2020). However, this is at odds with a recent study conducted by Kanatsu-Shinohara et al. that monitored long-term ‘ageing’ of SSCs in *vitro* culture (2019). In this study, authors reported an *increase* in glycolysis with prolonged culture time (comparing cultures maintained for 5 versus 60 months), which they associated with aberrant *Wnt7b* expression causing downstream effects on *Jnk* (*Mapk8*) and *Ppargc1a*. Unsurprisingly, by 60 months of *in vitro* culture, SSCs transplanted into recipient testes were no longer able to regenerate spermatogenesis, despite displaying hallmarks of glycolytic metabolism. There are several possible explanations for the difference in findings between this study and our own, the first being that Kanatsu-Shinohara et al. (2019) were not making comparisons between spermatogonia in culture versus those from the testis, but rather between two time points in culture. Indeed, the ‘young’ cultures in their study were maintained for 5 months *in vitro*, a time point at which a number of biological changes have already arguably occurred when compared to the original cell population retrieved from the testis (based on the significant decline in SSC content and regenerative capacity at this time (Helsel et al., 2017a)). Other differences include the age of the mice from which the spermatogonia were retrieved to establish cultures (adult versus postnatal), and the genetic background of the mice (C57BL6J versus DBA/2). Regardless, cumulatively these studies demonstrate that prolonged exposure to an *in vitro* environment exerts significant effects on SSC metabolism that negatively impairs their long-term regenerative capacity.

Of particular interest in considering the safety of using cultured SSCs for therapeutic purposes was the identification of downregulated DNA repair pathways in undifferentiated spermatogonia following 10 weeks of *in vitro* culture. For example, expression of the homology-directed repair enzymes *Brca2* and *Rad51* were found to be significantly reduced. In alignment with this, Kanatsu-Shinohara et al. (2019) found that markers of DNA damage were significantly increased in undifferentiated spermatogonia with prolonged culture time. Given the correlation between DNA damage in spermatozoa and male infertility (Aitken and Baker, 2020), it will be important to further investigate the link between SSC culture and DNA damage in the future, and the fate of “damaged” SSCs following their transplantation back into the testis.

One of the most striking findings of our investigation was the significant downregulated expression of a number of proteasomal subunits and other components of the ubiquitin-mediated proteolysis pathway in undifferentiated spermatogonia following 10 weeks in culture. The consequences of this loss of expression on proteasome function could be appreciated by the pronounced accumulation of ubiquitinated proteins observed in undifferentiated spermatogonia from culture when compared to those retrieved directly from the testis. Although it is well established that homeostasis between the synthesis and degradation of proteins (proteostasis) is integral for male fertility (Cafe et al., 2021a), little is known about requirements for proteostasis in the SSC population. Intriguingly however, in hematopoietic stem cells, proteostasis has been shown to be paramount for proper function, with the accumulation of misfolded proteins directly impairing self-renewal capacity and stem cell quiescence (Hidalgo San Jose et al., 2020). This raises the possibility that a similar mechanism may exist in SSCs, and that proteasome dysfunction with prolonged culture time could be linked to a loss of self-renewal capacity (Helsel et al., 2017a). Also worth noting is the propensity for oxidative stress to instigate protein unfolding/misfolding and subsequent protein aggregation (Mihalas et al., 2018; Nixon et al., 2019; Cafe et al., 2021a). In considering that a network of genes related to ‘oxidative phosphorylation’ were upregulated in cultured spermatogonia within our analyses, it is possible that this, in concert with dysregulated function of the ubiquitin-mediated proteolysis pathway, creates the ‘perfect storm’ for accumulation of misfolded proteins/protein aggregates.

In conclusion, prolonged culture of SSCs undoubtedly instigates a range of biological changes that negatively impact the cells capacity for regeneration. Regardless, primary cultures of undifferentiated spermatogonia remain an invaluable tool for discovery research into molecular regulation of SSC function, and hold great promise as a component of therapeutic pipelines to restore fertility, or for conservation purposes. In this manuscript we have provided the first in-depth analysis of changes in gene expression in undifferentiated spermatogonia following a period of culture, which will be an instrumental resource for developing this technique into the future.

## Data Availability

Publicly available datasets were analyzed in this study. These data can be accessed from the GEO database: GSE109033—adult spermatogonia database, and GSE163027—spermatogonia following in vitro culture.
