# Supplementary material for: A scRNA-seq Approach to Identifying Changes in Spermatogonial Stem Cell Gene Expression Following in vitro Culture
Source: Front Cell Dev Biol. 2022 Apr 1;10:782996. doi: 10.3389/fcell.2022.782996 (PMC9010880; doi:10.3389/fcell.2022.782996)
Supplement: Supplementary file 1 [file DataSheet1.pdf]

Supplementary Material

1.1 Supplementary Figures

Supplemental Figure 1

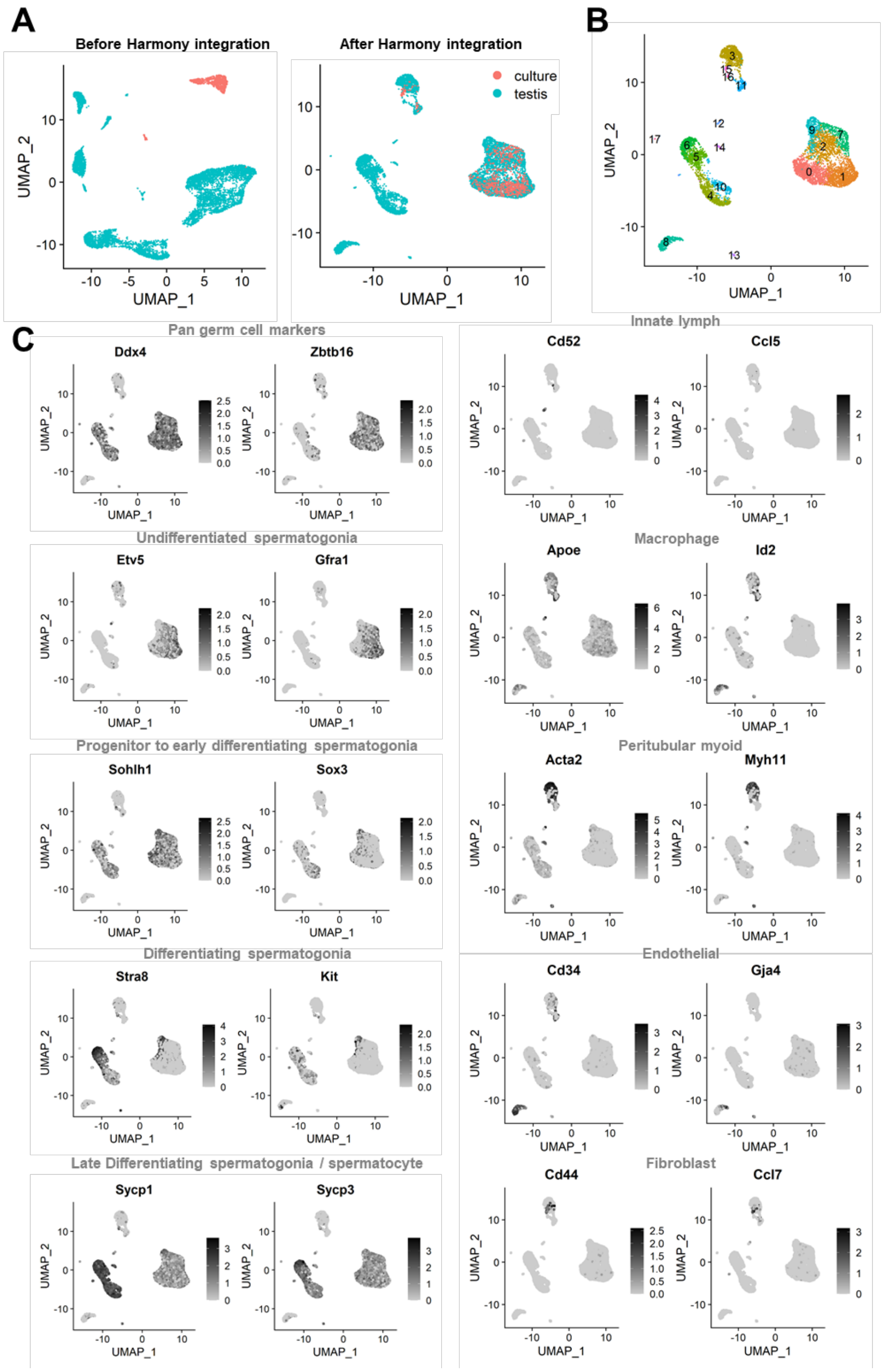

Supplemental Figure 1: Additional scRNA-seq analyses for combined germ cell and somatic cell dataset. (A) UMAP plots depicting the distribution of cells from culture and testis datasets before and after integration using the Harmony algorithm. **(B)** UMAP plot showing 18 clusters, which were re-classified into 9 distinct cellular populations (Fig. 1A) based on the expression of known markers. **(C)** Feature plots showing expression of known markers for germ cells (*Ddx4, Zbtb16*), undifferentiated spermatogonia (*Etv5, Gfra1*), progenitor / early differentiating spermatogonia (*Sohlh1, Sox3*), differentiating spermatogonia (*Kit, Stra8*), late differentiating spermatogonia / spermatocytes (*Sycp1, Sycp3*), innate lymph (*Cd52, Ccl5*), macrophages (*Apoe, Id2*), peritubular myoid cells (*Myh11, Acta2*), endothelial cells (*Gja4, Cd34*), and fibroblast feeder cells carried over from the culture dataset (*Cd44, Ccl7*).

Supplemental Figure 2

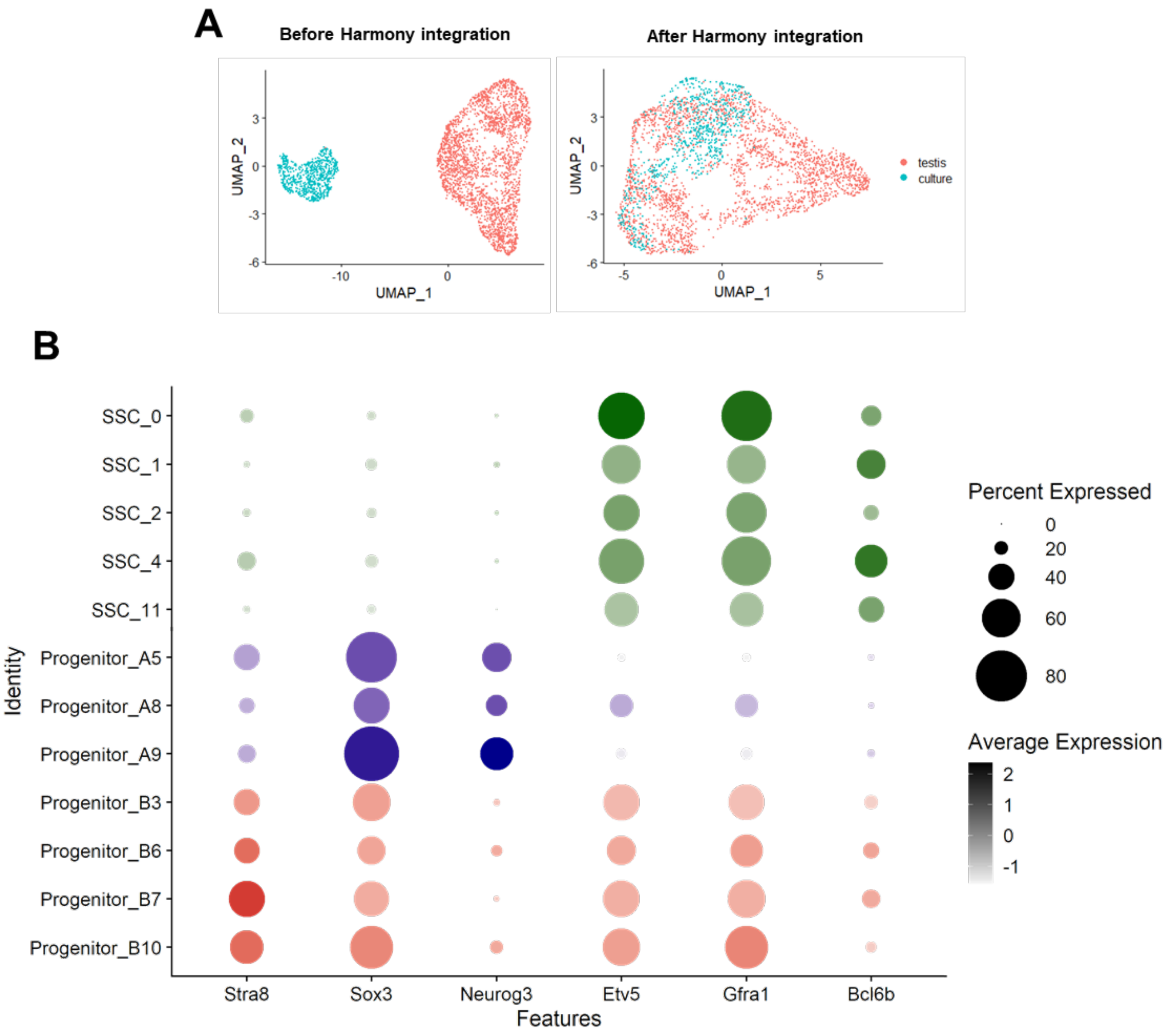

Supplemental Figure 2: Additional scRNA-seq analyses for undifferentiated spermatogonia dataset. (A) UMAP plots depicting the distribution of undifferentiated spermatogonia from culture and testis datasets before and after integration using the Harmony algorithm. **(B)** Dot plot showing expression of known markers for SSCs (*Bcl6b, Gfra1, Etv5*), progenitors (*Neurog3, Sox3*), and differentiating spermatogonia (*Stra8*). Dot size is percentage of cells expressing each gene, dot colour is level of expression. Accompanying feature plots are present in Fig. 1E. Violin plots depicting expression of additional SSC genes are provided in Fig. 2C.

Supplemental Dataset 1: Unique cluster markers and differentially expressed gene lists produced from scRNA-seq analysis.
